# Differential Diagnosis of Crohn’s Disease and Ulcerative Primary Intestinal Lymphoma: A Scoring Model Based on a Multicenter Study

**DOI:** 10.3389/fonc.2022.856345

**Published:** 2022-05-02

**Authors:** Hong Yang, Huimin Zhang, Wei Liu, Bei Tan, Tao Guo, Xiang Gao, Rui Feng, Kaichun Wu, Qian Cao, Zhihua Ran, Zhanju Liu, Naizhong Hu, Liangru Zhu, Yamin Lai, Congling Wang, Wei Han, Jiaming Qian

**Affiliations:** ^1^ Department of Gastroenterology, Peking Union Medical College Hospital, Chinese Academy Medical Sciences and Peking Union Medical College, Beijing, China; ^2^ Department of Radiology, Peking Union Medical College Hospital, Chinese Academy Medical Sciences and Peking Union Medical College, Beijing, China; ^3^ Department of Gastroenterology, The Sixth Affiliated Hospital of Sun Yat-sen University, Guangzhou, China; ^4^ Department of Gastroenterology, The First Affiliated Hospital of Sun Yat-sen University, Guangzhou, China; ^5^ State Key Laboratory of Cancer Biology, National Clinical Research Center for Digestive Diseases and Xijing Hospital of Digestive Diseases, Fourth Military Medical University, Xi’an, China; ^6^ Department of Gastroenterology, Sir Run Run Shaw Hospital, College of Medicine Zhejiang University, Hangzhou, China; ^7^ Division of Gastroenterology and Hepatology, Key Laboratory of Gastroenterology and Hepatology, Ministry of Health; Shanghai Inflammatory Bowel Disease Research Center; Renji Hospital, School of Medicine, Shanghai Jiao Tong University, Shanghai, China; ^8^ Department of Gastroenterology, The Shanghai Tenth People’s Hospital, Tongji University, Shanghai, China; ^9^ Department of Gastroenterology, The First Affiliated Hospital of Anhui Medical University, Hefei, China; ^10^ Department of Gastroenterology, Wuhan Union Hospital, Tongji Medical College, Huazhong University of Science and Technology, Wuhan, China; ^11^ Department of Epidemiology and Biostatistics, Institute of Basic Medical Sciences, Peking Union Medical College, Chinese Academy of Medical Sciences, School of Basic Medicine, Beijing, China

**Keywords:** Crohn’s disease, ulcerative primary intestinal lymphoma, diagnosis, imaging, scoring model

## Abstract

**Background:**

Differential diagnosis of Crohn’s disease (CD) and ulcerative primary intestinal lymphoma (UPIL) is a tough problem in clinical practice.

**Aims:**

Our study identified key differences between CD and UPIL patients and aimed to further establish a scoring model for differential diagnosis.

**Methods:**

A total of 91 CD and 50 UPIL patients from 9 tertiary inflammatory bowel disease centers were included. Univariate and multivariate analyses were used to determine significant markers for differentiating CD and UPIL. A differential scoring model was established by logistic regression analysis.

**Results:**

The differential model was based on clinical symptoms, endoscopic and imaging features that were assigned different scores: intestinal bleeding (−2 points), extraintestinal manifestation (2 points), segmental lesions (1 point), cobblestone sign (2 points), homogeneous enhancement (−1 point), mild enhancement (−1 point), engorged vasa recta (1 point). A total score of ≥1 point indicates CD, otherwise UPIL was indicated. This model produced an accuracy of 83.66% and an area under the ROC curve of 0.947. The area under the ROC curve for validation using the 10-fold validation method was 0.901.

**Conclusion:**

This study provided a convenient and useful model to differentiate CD from UPIL.

## Highlights

What is known:

- CD and UPIL have different therapy and prognosis
- Differential diagnosis of CD and UPIL is difficult

What is new here

- Endoscopic and imaging indicators significantly improved the ability to differentiate between CD and UPIL
- This model in our study is useful to differentiate CD from UPIL.

## Introduction

Ileocolonic ulcers appear in many clinical conditions such as Crohn’s disease (CD), intestinal tuberculosis (ITB), and primary intestinal lymphoma (PIL) (1). CD is a chronic inflammatory disease that affects the whole gastrointestinal tract, especially the terminal ileum and ileocecal region. The incidence rate of CD has recently increased in China. PIL is a heterogeneous disease that can be classified as fungating, ulcerative, or other types according to endoscopic morphology (2). A fungating lesion is a warning sign for physicians to keep vigilant watch for potential malignancy, whereas ulcerative lesions are easily ignored. There are many similarities between CD and ulcerative primary intestinal lymphoma (UPIL), and some reports have suggested that UPIL can be easily misdiagnosed as CD (3). Since UPIL often requires intense sequential chemotherapy treatment and carries an unfavorable prognosis (4), and physicians should effectively differentiate CD from UPIL.

The gold diagnostic criteria for CD and UPIL rely on pathology, but it is difficult to obtain typical pathological manifestations through biopsy specimens for both diseases. A recent article reported a potential model for the differential diagnosis between CD and PIL (5). However, since all types of PIL were enrolled in this study, it is not conducive to summarize the characteristics of UPIL. Differential diagnosis for CD and UPIL is still a tough problem in clinical practice.

To enhance the differential diagnostic efficiency and reduce the misdiagnosis rate, we analyzed the key differences in the clinical, endoscopic, and imaging characteristics between CD and UPIL patients. Furthermore, we evaluated the diagnostic value of different markers and established a scoring model for differential diagnosis.

## Methods

### Patients

A total of 91 CD patients and 50 UPIL patients were enrolled in this study between 1 January 2004, and 30 December 2018. These patients were diagnosed and treated at nine centers, namely, the Peking Union Medical College Hospital, the Sixth Affiliated Hospital of Sun Yat-sen University, the First Affiliated Hospital of Sun Yat-sen University, the Xijing Hospital, the Sir Run Shaw Hospital, the Shanghai Renji Hospital, the Shanghai Tenth People’s Hospital, the Affiliated Hospital of Anhui Medical University, and the Wuhan Union Hospital. Clinical, endoscopic, and imaging data were collected from all patients. The Institutional Review Board of Peking Union Medical College Hospital (S-K1100) approved the study.

### Inclusion and Exclusion Criteria

The inclusion criteria are as follows: (1) clinically or pathologically confirmed CD or pathologically confirmed PIL; and (2) clinical, endoscopic, and radiographic data were available for the majority of the patients. Endoscopic and imaging data include the original images and reports. For each item, the proportion of missing cases was lower than 20%.

The exclusion criteria are as follows: (1) PIL with fungating or other non-ulcerative lesions; and (2) PIL with post-surgery or treatment data (patients with data before the surgery or treatment could be included).

### Diagnostic Criteria

All patients were diagnosed with CD according to the European Crohn’s and Colitis Organization (ECCO) guidelines and Chinese consensus based on clinical manifestations, endoscopic features, and imaging or pathological features (6, 7).

All patients were diagnosed with PIL by histological results according to Dawson’s criteria (8).

### Data Collection

#### Demographic and Clinical Data

Demographic and clinical data included the sex, age at the onset of gastrointestinal symptoms, clinical manifestations, intestinal complications, extraintestinal manifestations (EIMs) (oral and vulvar ulcers, skin lesions, joint lesions, ocular lesions, fatty liver, cholelithiasis, thromboembolic disease, and myelodysplastic syndromes), past, and personal history of the patient (shown in **Tables 1**, **2**). Skin lesions are mainly referred to as nodular erythema, pyoderma gangrenosum, pseudofolliculitis, papules, and acne-like nodules, and are diagnosed by dermatologists. Ocular lesions mainly include uveitis, iritis, scleritis, and retinal vasculitis, and are diagnosed by ophthalmologists.

**Table 1:** Demographic Characteristics of participants with Crohn’s disease or Ulcerative Primary Intestinal Lymphoma.

Characteristics	UPIL (n = 50)	CD (n = 91)	Regression coefficient	OR (95% CI)	P*
Gender, Male, n (%)	36 (72.0%)	68 (74.7%)	−0.14	0.87 (0.40–1.92)	0.73
Onset age, mean (SD), y	45.62 ± 18.35	28.12 ± 12.08	−0.07	0.93 (0.90–0.95)	<0.001

CD, Crohn’s disease; UPIL, Ulcerative Primary intestinal lymphoma.

*Univariate logistic regression is used.

**Table 2:** Clinical Characteristics of Participants with Crohn’s disease or Ulcerative Primary Intestinal Lymphoma.

Characteristics	UPIL (n = 50)	CD (n = 91)	Regression coefficient	OR (95% CI)	P*
Clinical Manifestations, n (%)					
Fever	28 (56.0%)	39 (42.86%)	−0.52	0.59 (0.29–1.18)	0.14
Nausea	5 (10.0%)	20 (21.98%)	0.93	2.54 (0.95–8.05)	0.08
Abdominal Pain	41 (82.0%)	80 (87.91%)	0.47	1.60 (0.60–4.17)	0.34
Diarrhea	28 (56.0%)	62 (68.13%)	0.52	1.68 (0.82–3.43)	0.15
Hematochezia	21 (42.0%)	28 (30.77%)	−0.49	0.61 (0.30–1.26)	0.18
Perianal lesions	0	42 (46.15%)	18.59		0.99
Anorexia	22 (44.0%)	27 (29.67%)	−0.62	0.54 (0.26–1.10)	0.09
Weight loss	36 (72.0%)	62 (68.13%)	−0.18	0.83 (0.38–1.76)	0.63
Abdominal mass	8 (16.0%)	9 (9.89%)	−0.55	0.58 (0.21–1.64)	0.29
Onset Symptoms, n (%)					
Abdominal pain	32 (64.0%)	53 (58.24%)	−0.24	0.78 (0.38–1.59)	0.50
Diarrhea	26 (52.0%)	46 (50.55%)	−0.06	0.94 (0.47–1.88)	0.87
Perianal lesions	1 (2.0%)	18 (19.78%)	2.49	12.08 (2.37–220.89)	0.02
Complications, n (%)					
Abdominal abscess	2 (4.00%)	5 (5.49%)	0.33	1.40 (0.29–10.00)	0.70
Intestinal fistulas	5 (10.00%)	15 (16.48%)	0.57	1.78 (0.64–5.76)	0.30
Intestinal stenosis	9 (18.00%)	41 (45.05%)	1.32	3.74 (1.68–9.01)	0.002
Intestinal obstruction	9 (18.00%)	18 (19.78%)	0.12	1.12 (0.47–2.83)	0.80
Intestinal perforation	14 (28.00%)	1 (1.10%)	−3.56	0.03 (0.002–0.15)	<0.001
Intestinal bleeding	15 (30.00%)	7 (7.69%)	−1.64	0.19 (0.07–0.50)	0.001
EIMs	7 (14.00%)	35 (38.46%)	1.35	3.84 (1.63–10.18)	0.004
History of appendectomy	5 (10.00%)	12 (13.19%)	0.31	1.37 (0.47–4.52)	0.58
Smoking, n (%)	19 (38.00%)	23 (25.27%)	−0.59	0.55 (0.26–1.16)	0.12
Drinking, n (%)	16 (32.00%)	13 (14.29%)	−1.04	0.35 (0.15–0.81)	0.01

CD, Crohn’s disease; UPIL, Ulcerative Primary intestinal lymphoma; EIMs, extraintestinal manifestations.

*Univariate logistic regression is used.

#### Endoscopic Data

**Figure 1:**
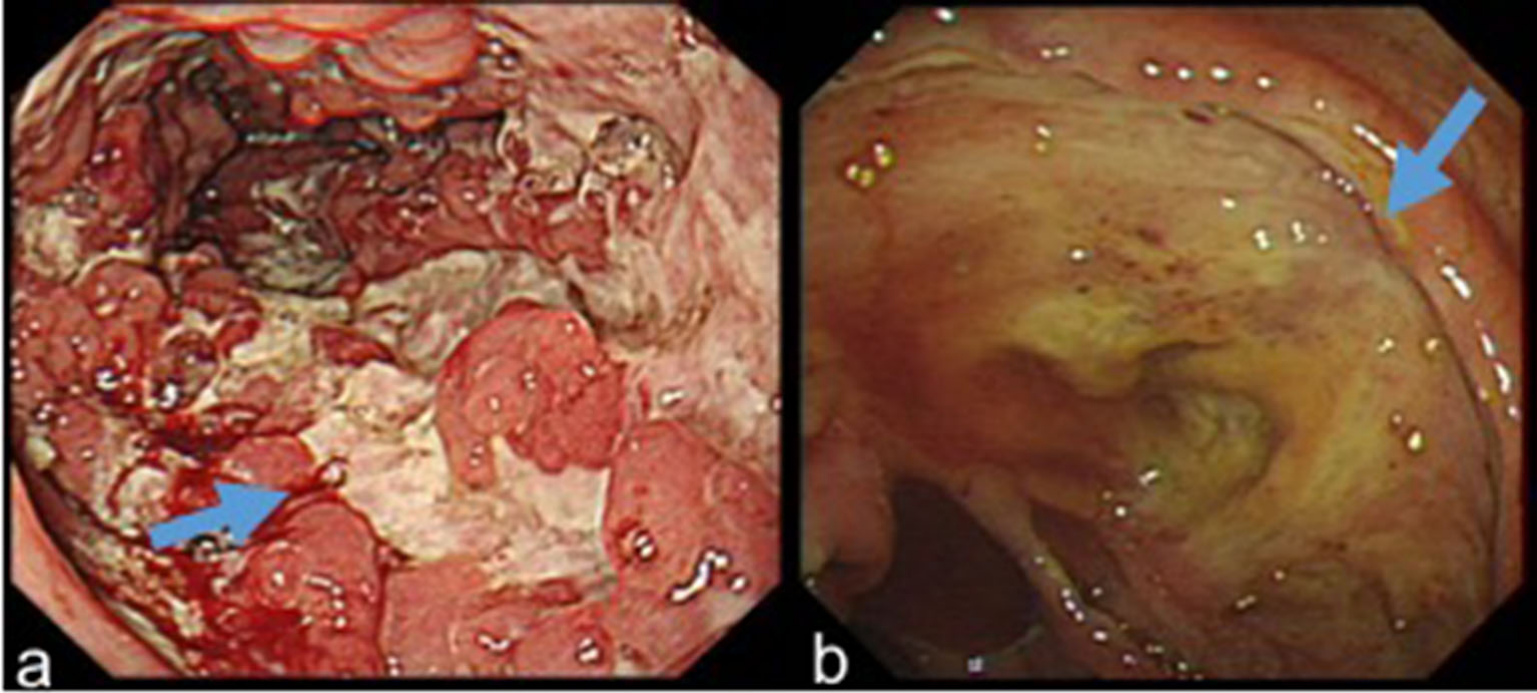
**(A)** Longitudinal ulcers, inflammatory polyps and cobblestone sign in a patient with CD. **(B)** A large and deep ulcer in a patient with UPIL.

Endoscopic indicators included lesion locations, segmental lesions, ulcer morphology (shallow, deep, longitudinal, irregular, annular, oval, and aphthous ulcers), number of ulcers (1, 2–5, and >5), ulcer diameter (<5 mm, 5–20 mm, and >20 mm), inflammatory polyps, mucosal bridges, and cobblestone signs (**Figure 1**). The definitions of the above variables were taken from the articles published by Li and He (9, 10).

Two experienced endoscopic physicians independently read all the endoscopic data. If their opinions were inconsistent, the final diagnosis was made after discussion.

#### Imaging Data

**Figure 2:**
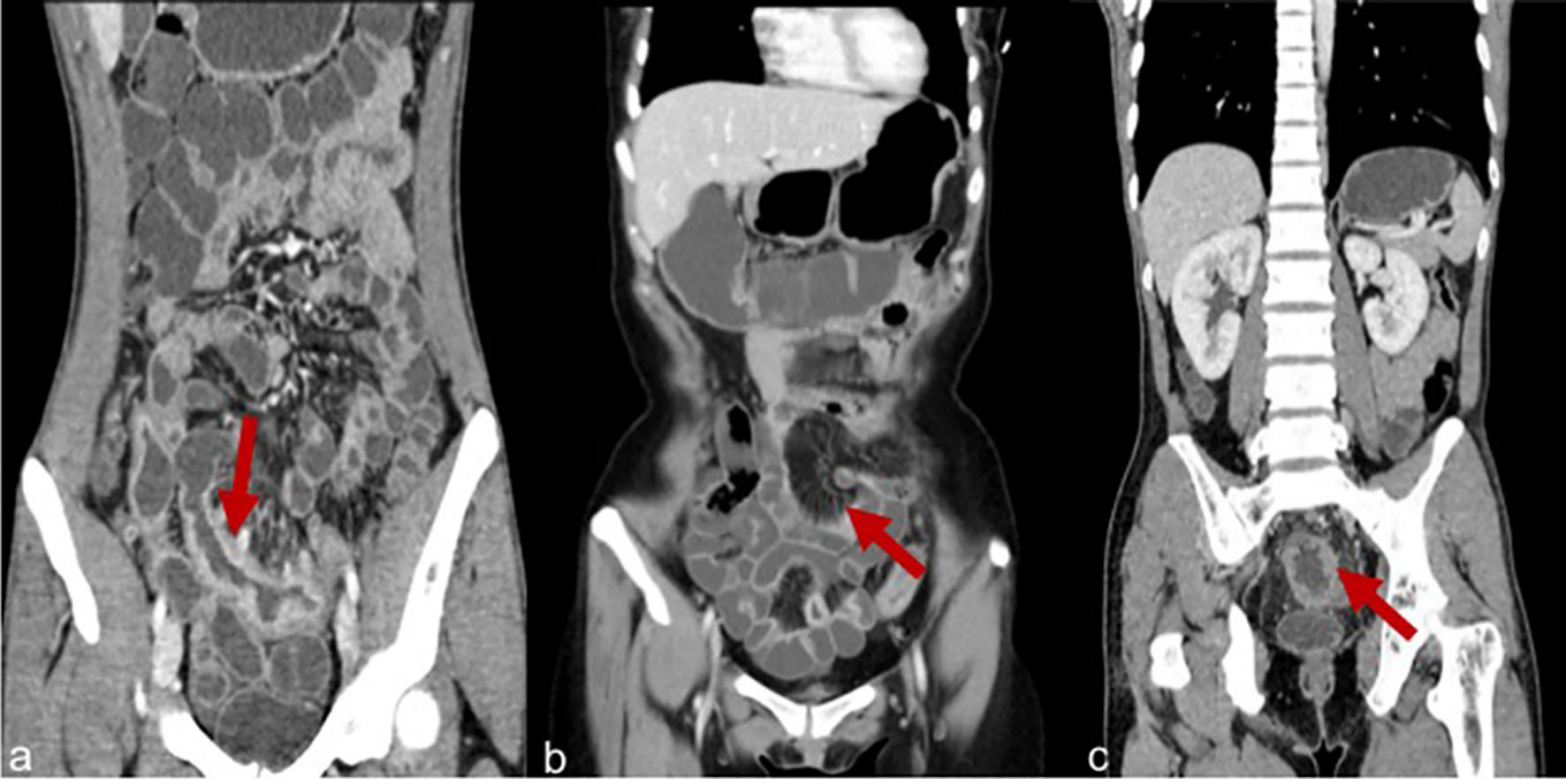
**(A)** Severe enhancement in a patient with CD. **(B)** Engorged vasa recta in a patient with CD. **(C)** Homogeneous enhancement in a patient with CD.

Imaging items included the length of the longest lesion segment, the thickness of the lesion, enhancement degree, homogeneous hyperenhancement, asymmetric mural hyperenhancement, polypoid lesion of the mucosal surface, fibrofatty proliferation, and engorged vasa recta (**Figure 2**). The definition of the above variables refers to the article published by Guglielmo (11).

Two radiologists independently reviewed all the imaging data. When their opinions were inconsistent, the final diagnosis was made after discussion.

#### Statistical Analysis

Continuous variables following an apparently normal distribution were summarized by the mean [standard deviation (SD)]; otherwise, they were summarized by the median and interquartile range. Categorical variables were presented as proportions. Univariate analysis was conducted using logistic regression with one independent variable. Receiver operating characteristic (ROC) curve analysis was used to determine the threshold value for continuous variables presenting a linear assumption, otherwise Lowess smoothing function was used. A multivariate logistic regression model was built after univariate analysis, and a further variable selection procedure was conducted, and a final multivariate model was developed by incorporating variables with statistical significance (P-value <0.05) variables. Variables with marginal statistical significance (P-value slightly >0.05) were also included in the final multivariate model due to their clinical significance.

A scoring system was built based on the final multivariate model. Taking the variable with the minimum regression coefficient as 1 point, the scores of other variables were obtained by dividing their regression coefficients with the minimum regression coefficient and rounding to the nearest integer. The Youden Index was used to determine the cutoff value of the scoring model. The total risk score of each patient was the sum of all the scores of predictors assigned to him or her.

Evaluating the predictive performance (AUC) of the fitted model using all cases from the original analysis sample tends to result in an overly optimistic estimate of the performance. Therefore, a 10-fold cross-validation procedure was employed to calculate a more realistic estimate of predictive performance (12). To calculate the 10-fold cross-validation AUC, the original data set was randomly divided into ten parts, and the first tenth of the data was held out as a validation set, with a logistic model being fitted using the remaining observations. Then, the predicted probability for each observation in the validation set was calculated using the training model. This procedure was repeated 10 times, and an AUC score was calculated for each of the 10 runs, and then the average AUC was calculated.

All the data were analyzed by SAS9.2 (SAS Institute, Inc, Cary, NC).

## Results

### Demographic Characteristics

Among the 50 UPIL patients, 17 lymphomas (34%) were of B-cell origin, and the rest of the cases (33 cases, 66%) were of NK-cell and T-cell origins. The details are shown in **Table 3**.

**Table 3:** Subtypes of Ulcerative Primary intestinal lymphoma.

Subtypes of lymphoma	Number of Cases
B-cell Origin	17
Diffuse large B cell lymphoma	5
Mantle cell lymphoma	2
Mucosa associated lymphoid tissue lymphoma	2
Plasmablastic lymphoma	1
Burkitt lymphoma	1
Hodgkin’s lymphoma	1
Unclassified B-cell lymphoma	5
NK/T-cell Origin	33
NK/T cell lymphoma	17
T-cell lymphoma	14
Enteropathy associated T-cell lymphoma	2

As shown in **Table 1**, there was no significant difference in gender between the CD and UPIL patients. The age of onset of gastrointestinal symptoms in UPIL was significantly higher than that in CD patients (P <0.001).

### Univariate Analysis to Compare the Clinical Characteristics Between CD and UPIL

#### Comparative Analysis of Clinical Symptoms

The clinical data (**Table 2**) showed that the incidence rates of intestinal stenosis and EIMs were significantly higher in patients with CD than in those with UPIL (all P <0.05). In contrast, the incidence rates of intestinal perforation and bleeding were significantly lower in CD patients compared to UPIL patients (both P <0.05).

#### Comparative Analysis of Endoscopic Characteristics

The endoscopic characteristics (**Table 4**) showed that for lesion location(s), the proportion of the ileocecal and ascending colon involvement in patients with CD was significantly higher than in patients with UPIL (both P <0.05). Regarding the distribution of lesions, patients with CD were also more likely to have segmental lesions (P <0.001). In terms of ulcer morphology, the proportions of patients with CD who had shallow and longitudinal ulcers were higher than those of patients with UPIL (both P <0.05). Furthermore, patients with UPIL are more likely to have a single and large ulcer with a diameter exceeding 20 mm compared to CD (P <0.05). Additionally, the proportions of inflammatory polyps, mucosal bridges, and cobblestone signs in patients with CD were higher (P <0.05).

**Table 4:** Endoscopic Characteristics of Participants with Crohn’s disease or Ulcerative Primary Intestinal Lymphoma.

Variables	UPIL (n = 50)	CD (n = 91)	Regression coefficient	OR (95% CI)	P*
Lesion Site					
Ileocecal region	18 (36.00%)	65 (71.43%)	1.49	4.44 (2.16–9.44)	<0.001
Ascending colon	14 (28.00%)	42 (46.15%)	0.79	2.20 (1.07–4.74)	0.04
Transverse colon	14 (28.00%)	38 (41.76%)	0.61	1.84 (0.89–3.97)	0.11
Descending colon	15 (30.00%)	32 (35.16%)	0.24	1.27 (0.61–2.70)	0.53
Sigmoid colon	15 (30.00%)	39 (42.86%)	0.56	1.75 (0.85–3.71)	0.13
Rectum	10 (10.00%)	24 (26.37%)	0.36	1.43 (0.63–3.42)	0.40
Segmental Lesions	20 (40.00%)	68 (74.73%)	1.49	4.43 (2.15–9.42)	<0.001
Ulcer Morphology					
Shallow ulcer	8 (16.33%)	30 (32.97%)	0.92	2.52 (1.09–6.39)	0.04
Deep ulcer	35 (71.43%)	51 (56.04%)	−0.67	0.51 (0.24–1.06)	0.08
Longitudinal ulcer	8 (16.33%)	35 (38.46%)	1.16	3.20 (1.40–8.08)	0.009
Irregular ulcer	26 (53.06%)	60 (65.93%)	0.54	1.71 (0.84–3.49)	0.14
Annular ulcer	7 (14.29%)	4 (4.40%)	−1.29	0.28 (0.07–0.96)	0.05
Oval ulcer	3 (6.12%)	9 (9.89%)	0.52	1.68 (0.47–7.87)	0.45
Aphtha	4 (8.16%)	13 (14.29%)	0.63	1.88 (0.62–6.97)	0.30
Number of Ulcers					
1	21 (42.86%)	19 (20.88%)	–	–	–
2–5	12 (24.49%)	23 (25.27%)	0.75	2.12 (0.84–5.50)	0.12
>5	16 (32.65%)	49 (53.85%)	1.22	3.38 (1.48–7.97)	0.004
Ulcer Diameter					
<5 mm	4 (8.33%)	12 (13.19%)	–	–	–
5–20 mm	7 (14.58%)	50 (54.95%)	0.87	2.38 (0.55–9.30)	0.22
>20 mm	37 (77.08%)	29 (31.87%)	−1.34	0.26 (0.07–0.84)	0.03
Lymphangiectasia	5 (10.20%)	0	−17.29	–	0.99
Inflammatory Polyps	6 (12.24%)	41 (45.05%)	1.77	5.88 (2.42–16.61)	<0.001
Mucosal Bridge	0	10 (10.99%)	−1.99	0.14 (0.05-0.36)	<0.001
Cobblestone Appearance	1 (2.04%)	27 (29.67%)	3.01	20.25 (4.08-367.49)	<0.001

CD, Crohn’s disease; UPIL, Ulcerative Primary intestinal lymphoma.

*Univariate logistic regression is used.

#### Comparative Analysis of Imaging Characteristics

The imaging data (**Table 5**) showed that patients with CD had significantly longer but thinner lesions than UPIL patients (P <0.05). In terms of lesion enhancement characteristics, most patients with UPIL displayed mild enhancement, while most CD patients displayed moderate to severe enhancement. CD patients are more prone to asymmetric mural hyperenhancement, while UPIL patients are more likely to have homogeneous enhancement. Additionally, compared to patients with UPIL, patients with CD were more prone to mucosal polypoid bulges, fibrofatty proliferation, and engorged vasa recta (all P <0.05).

**Table 5:** Imaging Characteristics of Participants with Crohn’s disease or Ulcerative Primary Intestinal Lymphoma.

Variables	UPIL (n = 50)	CD (n = 91)	Regression coefficient	OR (95%CI)	P*
Segment Lesion Length					
<5 cm	12 (26.09%)	16 (17.58%)	–	–	–
5–10 cm	22 (47.83%)	25 (27.47%)	−0.16	0.85 (0.33–2.18)	0.74
10–30 cm	7 (15.22%)	27 (29.67%)	1.06	2.89 (0.97–9.25)	0.06
>30 cm	5 (10.87%)	23 (25.27%)	1.24	3.45 (1.06–12.67)	0.05
Lesion Thickness <1 cm	19 (42.22%)	64 (70.33%)	1.18	3.24 (1.56–6.91)	0.002
Enhancement Degree					<
Mild	32 (69.57%)	17 (18.68%)	–	–	0.001
Moderate to severe	14 (30.43%)	74 (81.32%)	2.30	9.95 (4.49–23.29)	
Asymmetric Mural Hyperenhancement	9 (20.45%)	41 (45.05%)	−1.62	0.20 (0.09–0.41)	<0.001
Homogeneous Enhancement	30 (68.18%)	9 (9.89%)	2.97	19.52 (7.97–52.42)	<0.001
Polypoid Bulging of Mucosal Surface	7 (15.91%)	59 (64.84%)	2.28	9.75 (4.10–26.15)	<0.001
Fibrofatty Proliferation	18 (38.30%)	56 (61.54%)	0.95	2.58 (1.26–5.39)	0.01
Engorged Vasa Recta	10 (21.28%)	63 (69.23%)	2.12	8.33 (3.75–19.90)	<0.001

CD, Crohn’s disease; UPIL, Ulcerative Primary intestinal lymphoma.

*Univariate logistic regression is used.

### The Diagnostic Value of Different Indicators in CD and UPIL

To comparatively analyze the diagnostic value of clinical symptoms and various endoscopic and imaging indicators in CD and UPIL, we performed multivariate and ROC curve analyses of the clinical symptoms individually, clinical symptoms combined with endoscopic indicators, and clinical symptoms combined with endoscopic and imaging indicators to select the model with the best differential diagnostic power.

**Figure 3:**
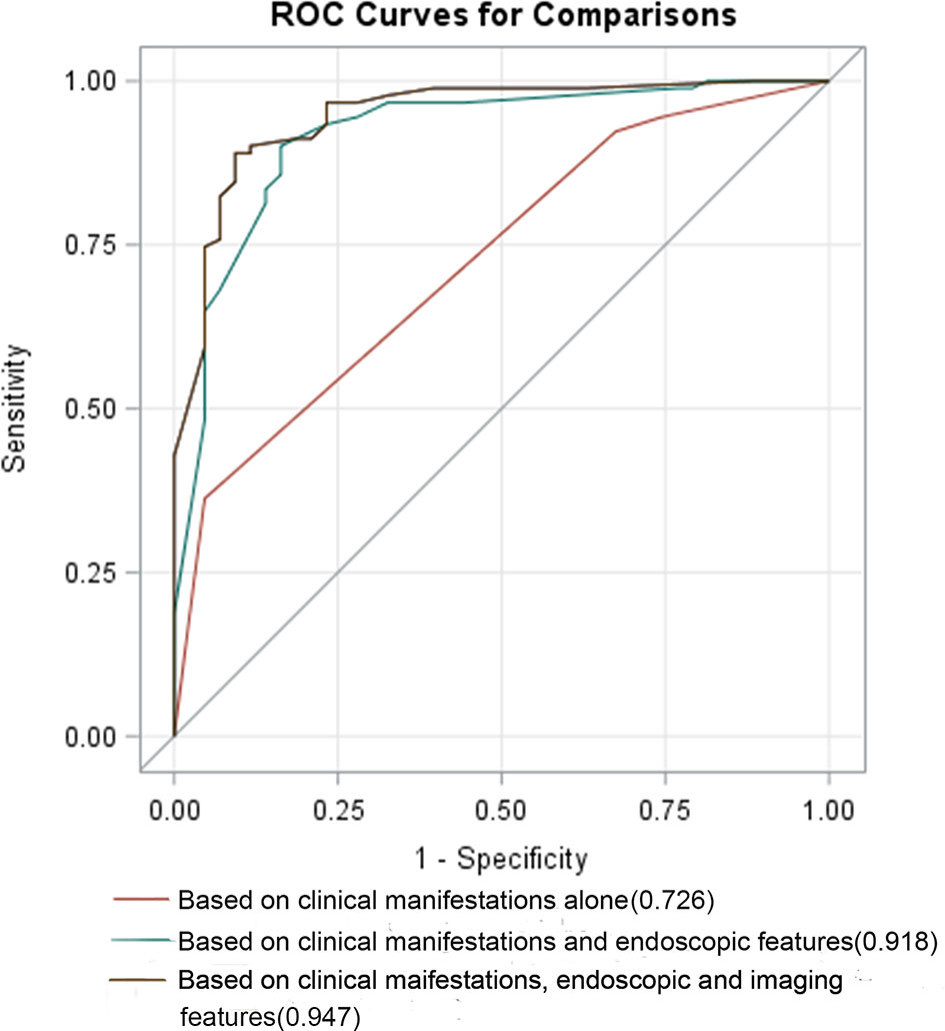
ROC curves based on different variables. The AUC for differentiating CD and UPIL based on clinical manifestations alone, combined clinical manifestations and endoscopic features, combined clinical manifestations, endoscopic, and imaging features was 0.726, 0.918, and 0.947, respectively.

First, we included clinical symptom indicators that produced P <0.05 with the univariate analysis in the multivariate logistic regression analysis. We found that the indicators with statistical significance were intestinal bleeding and EIMs. The AUC for differentiating CD and UPIL based on these two indicators was 0.726. We then performed a multivariate logistic regression analysis of clinical and endoscopic indicators that produced a P <0.05 in the univariate analysis. These indicators included intestinal bleeding, EIMs, segmental lesions, and cobblestone signs. Based on these four indicators, the AUC for differentiating CD and UPIL based on these four indicators was 0.918. Finally, we performed a multivariate logistic regression analysis of clinical, endoscopic, and imaging indicators with a P <0.05 in the univariate analysis, which included intestinal bleeding, EIMs, segmental lesions, cobblestone signs, homogeneous enhancement, mild enhancement, and engorged vasa recta (**Table 6**). Based on these seven indicators, the AUC for differentiating CD and UPIL was 0.947 (**Figure 3**). Statistical analysis showed that the last AUC was significantly higher than the other two AUCs (P <0.05) (**Table 7**).

**Table 6:** Multivariate Analysis and Scores Based on Clinical Manifestations, Endoscopic, and Imaging Characteristics.

Variable	Regression coefficient	OR	95% CI	*P*	Score
**Clinical Manifestations**					
Intestinal bleeding	-2.3876	0.092	0.019–0.452	0.003	−2
Extraintestinal manifestations	2.0993	8.161	1.462–45.563	0.02	2
**Endoscopic Characteristics**					
Segmental lesion	1.7663	5.849	1.602–21.358	0.008	1
Cobblestone sign	2.5769	13.157	0.863–200.571	0.06	2
**Imaging Characteristics**					
Homogeneous enhancement	−1.6304	0.196	0.052–0.742	0.02	−1
Mild enhancement	−1.8204	0.162	0.041–0.643	0.01	−1
Engorged vasa recta	1.2979	3.662	1.062–12.621	0.04	1

**Table 7:** Comparisons among different indicators.

Indicators	AUC	Accuracy
M-1	0.726	0.6950
M-2	0.918	0.8014
M-3	0.947	0.8366
Multiple Comparisons of AUC	*P*	–
M-2 vs M-1	<0.001	–
M-3 vs M-2	0.001	–
M-3 vs M-1	<0.001	–

M-1, indicators including intestinal bleeding and extraintestinal manifestations.

M-2, indicators including intestinal bleeding, extraintestinal manifestations, segmental lesion and cobblestone sign.

M-3, indicators including intestinal bleeding, extraintestinal manifestations, segmental lesion, cobblestone sign, homogeneous enhancement, mild enhancement and engorged vasa recta.

Therefore, including endoscopic and imaging indicators significantly improved the ability to differentiate between CD and UPIL.

### Establishment of a Differential Diagnosis Scoring Model for CD and UPIL

**Figure 4:**
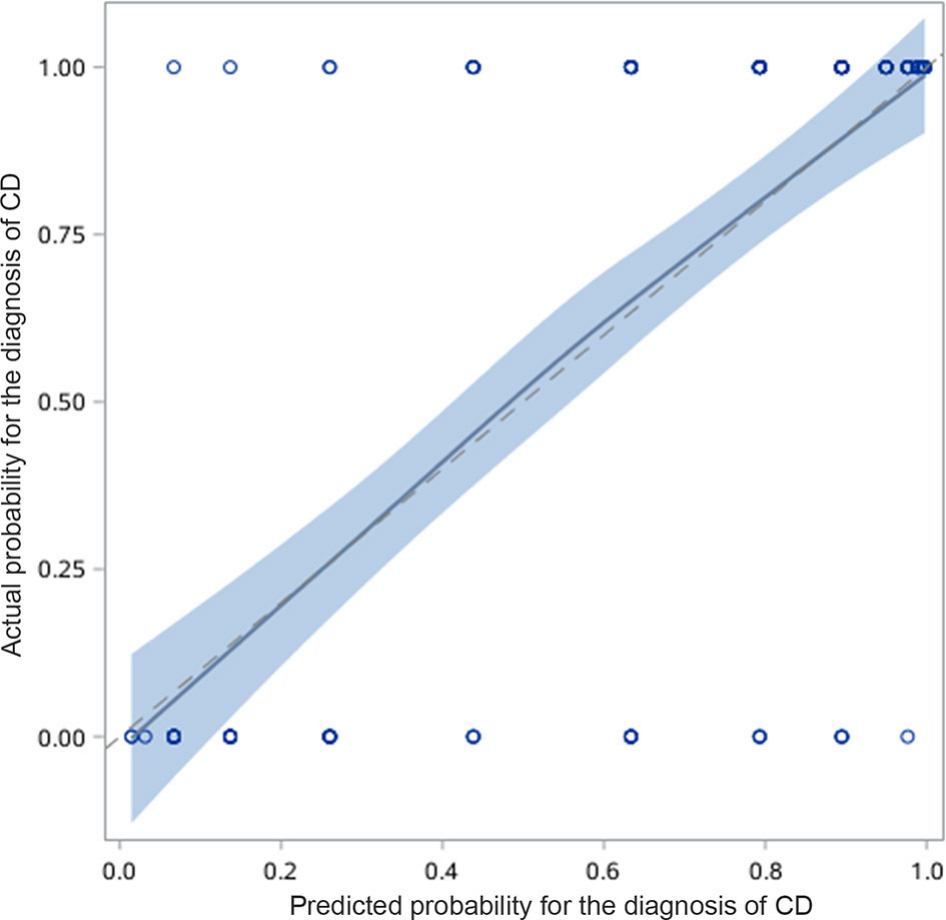
Calibration curve for predicting the possibility of CD. The calibration plot also demonstrated good performance of this score model.

The differential diagnostic scoring model for CD and UPIL was ultimately established based on the logistic regression model of clinical symptoms combined with endoscopic and imaging features. According to the previous multivariate analysis, we got the scores of each variable using the method shown in the statistical analysis subsection (**Table 6**). A patient was diagnosed with CD if the total score was ≥1; otherwise, he or she was diagnosed with UPIL. The accuracy of the differential diagnosis using this model was as high as 83.66%. The calibration plot also demonstrated the good performance of this score model (**Figure 4**).

### Validation of the Differential Diagnosis Scoring Model

**Figure 5:**
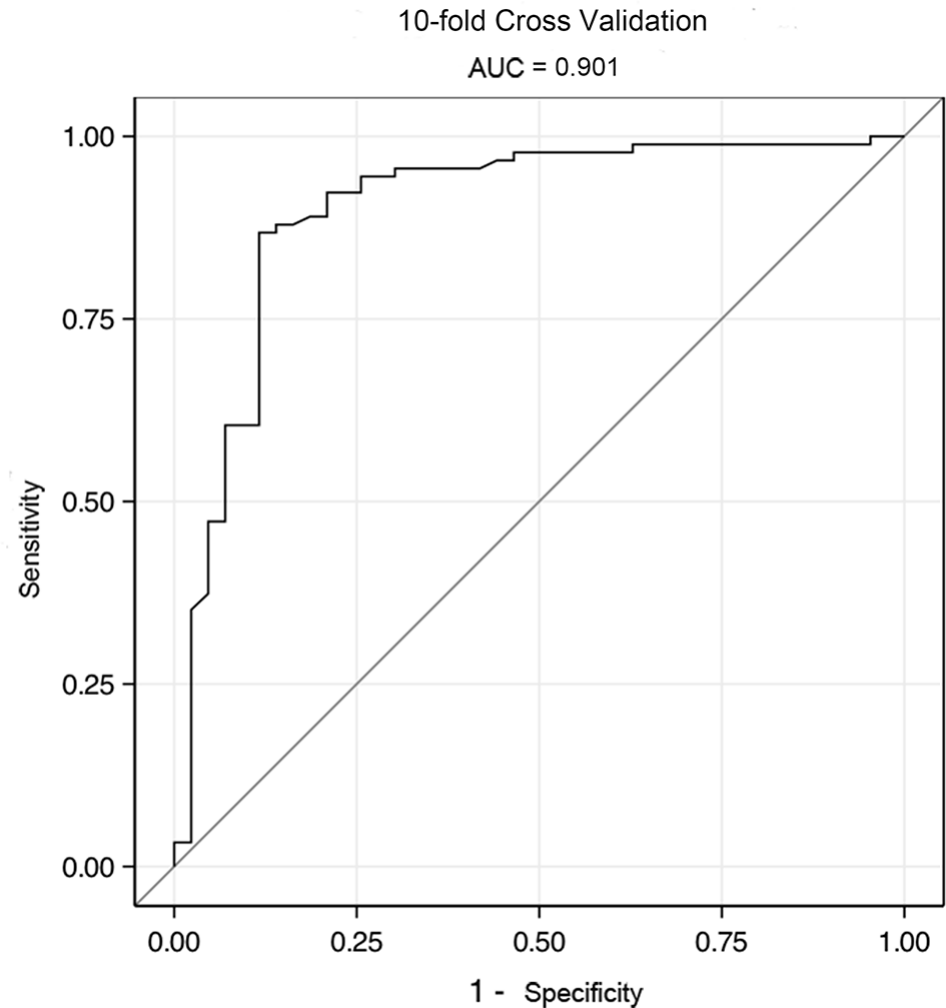
The ROC of validation model by 10-fold cross validation. The AUC for 10-fold validation was 0.901.

The above results were validated using a 10-fold validation method. The AUC for 10-fold validation was 0.901 (**Figure 5**), suggesting that this model can robustly differentiate between CD and UPIL.

## Discussion

Approximately 30 to 50% of extranodal lymphomas primarily involve the gastrointestinal tract, two-thirds of which are classified as ulcerative lesions (13). Pathology is the gold standard for the diagnosis of CD and UPIL. However, it is very difficult to obtain typical pathological manifestations and make a diagnosis through biopsy specimens. Granulomas are found only in 15–65% of the mucosal biopsy from patients with CD, while only 21% of intestinal non-Hodgkin’s lymphomas can be diagnosed by endoscopic biopsy (14, 15). Due to the lack of standardized diagnostic criteria for endoscopy features for CD and UPIL and the inherent limitations of endoscopic biopsies, 76.47% PIL patients were misdiagnosed as other diseases (16). Therefore, it is important for clinicians to be able to actively differentiate CD from UPIL.

In this study, we established a differential model using multivariate logistic regression that could robustly differentiate between CD and UPIL. This scoring model identified the most meaningful variables based on clinical symptoms and imaging and endoscopic characteristics. A total score ≥1 point indicates a diagnosis of CD, and less than 1 point indicates UPIL. Our predictive model produced an accuracy of 83.66% and an area under the ROC curve of 0.947, which will be conductive and helpful in clinical practice for differentiating between CD and UPIL.

In this study, demographic and clinical characteristics were compared between CD and UPIL patients. From univariate analyses, the results indicated that onset age, perianal lesions, intestinal perforation, intestinal bleeding, and EIMs appeared significantly different between CD and UPIL. It was significant that UPIL patients had an older age at onset, which was consistent with the results of the large-sample epidemiological studies of lymphoma (17). EIMs and perianal lesions were regarded as critical indicators of CD patients (18). Additionally, UPIL and CD can cause additional intestinal complications. However, more patients with CD than UPIL display intestinal stenosis, and more patients with UPIL than CD display intestinal perforation and hemorrhage. Sun et al. reported that 61.76% suffered from intestinal perforation and 2.94% from massive hematochezia in patients with intestinal T-cell lymphoma (16). The incidence of perforation is higher in UPIL. This may be due to the adherence of lymphoma cells to the vascular wall, which causes vascular occlusion, ischemic necrosis, and finally perforation.

Although endoscopic evaluation plays a key role in the diagnosis of CD and UPIL, there was no consensus on typical endoscopic features for UPIL; some cases depicted a diffuse or irregular ulcer that indicated UPIL, and others did not exhibit any specific characteristic (2, 13, 19). In univariate analyses, we found some different endoscopic features between these two groups, such as ulcer type, size quantity, pseudo-polyps, cobblestone appearance, and so on. Endoscopic features of these ulcers represent the different biological behaviors and histopathology of the two diseases. In multivariate analysis, the incidence rates of segmental distribution and cobblestone appearance were significantly different between CD and UPIL.

In terms of imaging, there are few comparative studies on CD and UPIL. Many studies show that the enhancement of gastrointestinal lymphoma is homogeneous, which mean it is equal to or lower in attenuation than the normal tissues (20). Necrosis of neoplasms may account for the low attenuation (21). For CD patients, Bodily et al. showed that mucosal surface hyperenhancement is highly correlated with histopathological activity (22). Moreover, mural stratification and engorged vasa recta are common in CD patients (23). An engorged vasa recta is also a specific manifestation of CTE in patients with active CD. However, this mechanism was not found in UPIL. The preservation of wall stratification is also a helpful CT criterion for differentiating benign from malignant diseases (24). Our multivariate analysis also showed that CD patients were more likely to have engorged vasa recta, while homogeneous and low attenuation were more common in UPIL patients. This result is similar to other studies (5, 25). As far as we are aware, this is the first study to compare imaging features of CD and UPIL, and we think this result is particularly interesting and useful as it provides a new method for differentiating CD and UPIL using imaging features.

To comparatively analyze the diagnostic value of clinical symptoms, endoscopic and imaging features in CD and UPIL, ROC curve analyses based on different multivariate analyses were performed. The AUCs for differentiating CD and UPIL were 0.726 based on clinical manifestations, 0.918 based on combined clinical manifestations combined with endoscopic features, and 0.947 based on the clinical manifestations combined with endoscopic and imaging features. Statistical analysis showed that the last AUC was significantly higher than the other two AUCs (P <0.05) (26). The results showed that endoscopic and imaging characteristics can improve the ability of clinical symptoms to differentiate between CD and UPIL.

Lastly, we scored the results of the multivariate analysis based on clinical symptoms, and endoscopic and imaging (CTE) data according to the coefficients of logistic regression analysis and established a diagnostic scoring model for CD and UPIL. The results suggest that the combination of clinical symptoms, CTE, and endoscopic features can robustly differentiate CD and UPIL. The AUC obtained from 10-fold cross-validation of this model was 0.901, indicating that it has strong predictive power for distinguishing CD and UPIL. It is worth noting that the indicator “cobblestone appearance” did not attain statistical significance in the scoring system. But the cobblestone sign is deemed the “colonoscopic marker” of CD, and it is visualized by multiple deep ulcers with elevated edematous mucosa interconnecting the ulcers. According to the published references, the cobblestone sign may not be very specific for CD, and it may also occur in other diseases like infection, but it is rare in UPIL. We therefore think the cobblestone sign is of great value in excluding UPIL. Furthermore, a label of statistical significance (P <0.05) does not mean or imply that an association or effect is highly probable, and many statisticians think that we should re-examine the meaning of P-value and move forward to a world beyond “P <0.05” (27). So we included this variable in the scoring system.

This is the first study to provide a combined clinical, endoscopic, and imaging-based model for differentiating UPIL from CD. However, the first notable limitation of this study is that there were various pathological types of lymphoma included, which could have affected the diagnostic model efficiency and the ultimate prediction accuracy. Secondly, 10-fold cross validation belongs to internal validation, and the objectivity is indeed inferior to external validation, which needs a larger sample size. We have tried our best to collect enough patients for external validation in this multicenter study. Unfortunately, UPIL is uncommon in clinical practice, and we did not collect enough patients to perform external validation. Because of the limited sample size, a ten-fold validation was performed, which verified the robustness of the model at present and had a lower variance than a single hold-out set estimator. However, it is still essential to further collect data for external verification, which is our future work plan; Thirdly, some markers like lactate dehydrogenase that are sensitive to the diagnosis of UPIL were excluded from this study. Fourthly, patients in the CD group suffered from a heavy level of activity restriction (moderate to severe), which may not represent the full spectrum of CD patients. Fifthly, this study was a retrospective with a small sample size, and future studies with larger populations must validate these results in full.

In conclusion, our results suggest that the combination of endoscopy, imaging features, and clinical symptoms is of great value for differentiating of CD and UPIL. The inclusion of more different subtypes of UPIL in the future will help establish a more accurate and meaningful differential model.

## Data Availability Statement

The raw data supporting the conclusions of this article will be made available by the authors, without undue reservation.

## Ethics Statement

Written informed consent was obtained from the individual(s), and minor(s)’ legal guardian/next of kin, for the publication of any potentially identifiable images or data included in this article.

## Author Contributions

HY and JQ: Study design, data collection and analysis support, and critical revision of manuscript. HZ: Data collection and analysis and drafting the manuscript. WL, BT, TG, XG, RF, KW, QC, ZR, ZL, NH, LZ, YL, and CW: Data collection. WH Data analysis. All authors listed have made a substantial, direct, and intellectual contribution to the work and approved it for publication.

## Funding

This work was supported by the National Nature Science Foundation of China [81570505 and 81970495], the Beijing Municipal Natural Science Foundation [7202161], the Health Research & Special Projects Grant of China [201502005], and the CAMS Innovation Fund for Medical Sciences (CIFMS) [2016-I2M-3-001 and 2019-I2M-2-007].

## Conflict of Interest

The authors declare that the research was conducted in the absence of any commercial or financial relationships that could be construed as a potential conflict of interest.

## Publisher’s Note

All claims expressed in this article are solely those of the authors and do not necessarily represent those of their affiliated organizations, or those of the publisher, the editors and the reviewers. Any product that may be evaluated in this article, or claim that may be made by its manufacturer, is not guaranteed or endorsed by the publisher.

## Abbreviations

CD, Crohn’s disease; UPIL, ulcerative primary intestinal lymphoma; ECCO, European Crohn’s and Colitis Organization; EIM, extraintestinal manifestations; SD, standard deviation; ROC, Receiver operating characteristic.

## References

[1] Lee JM Lee KM. Endoscopic Diagnosis and Differentiation of Inflammatory Bowel Disease. Clin Endosc (2016) 49(4):370–5. doi: 10.5946/ce.2016.090 PMC4977735 27484813

[2] Myung SJ Joo KR Yang SK Jung HY Chang HS Lee HJ. Clinicopathologic Features of Ileocolonic Malignant Lymphoma: Analysis According to Colonoscopic Classification. Gastrointest Endosc (2003) 57(3):343–7. doi: 10.1067/mge.2003.135 12612513

[3] Wu PH Chu KE Lin YM Huang SH Wu CC. T-Cell Lymphomas Presenting as Colon Ulcers and Eosinophilia. Case Rep Gastroenterol (2015) 9(2):246–52. doi: 10.1159/000437294 PMC4560296 26351412

[4] Lightner AL Shannon E Gibbons MM Russell MM. Primary Gastrointestinal Non-Hodgkin’s Lymphoma of the Small and Large Intestines: A Systematic Review. J Gastrointest Surg (2016) 20(4):827–39. doi: 10.1007/s11605-015-3052-4 26676930

[5] Zhang TY Lin Y Fan R Hu SR Cheng MM Zhang MC. Potential Model for Differential Diagnosis Between Crohn’s Disease and Primary Intestinal Lymphoma. World J Gastroenterol (2016) 22(42):9411–18. doi: 10.3748/wjg.v22.i42.9411 PMC5107705 27895429

[6] Gomollon F Dignass A Annese V Tilg H Van Assche G Lindsay JO. 3rd European Evidence-Based Consensus on the Diagnosis and Management of Crohn’s Disease 2016: Part 1: Diagnosis and Medical Management. J Crohns Colitis (2017) 11(1):3–25. doi: 10.1093/ecco-jcc/jjw168 27660341

[7] Inflammatory Bowel Disease Group Chinese Society Of Gastroenterology. Consensus on Diagnosis and Treatment of Inflammatory Bowel Disease (2018, Beijing). Chin J Dig (2018) 5:292–311.

[8] Dawson IM Cornes JS Morson BC. Primary Malignant Lymphoid Tumours of the Intestinal Tract. Report of 37 Cases With a Study of Factors Influencing Prognosis. Br J Surg (1961) 49:80–9. doi: 10.1002/bjs.18004921319 13884035

[9] Li J Li P Bai J Lyu H Li Y Yang H. Discriminating Potential of Extraintestinal Systemic Manifestations and Colonoscopic Features in Chinese Patients With Intestinal Behcet’s Disease and Crohn’s Disease. Chin Med J (Engl) (2015) 128(2):233–8. doi: 10.4103/0366-6999.149213 PMC4837844 25591568

[10] He Y Zhu Z Chen Y Chen F Wang Y Ouyang C. Development and Validation of a Novel Diagnostic Nomogram to Differentiate Between Intestinal Tuberculosis and Crohn’s Disease: A 6-Year Prospective Multicenter Study. Am J Gastroenterol (2019) 114(3):490–9. doi: 10.14309/ajg.0000000000000064 30741735

[11] Guglielmo FF Anupindi SA Fletcher JG Al-Hawary MM Dillman JR Grand DJ. Small Bowel Crohn Disease at CT and MR Enterography: Imaging Atlas and Glossary of Terms. Radiographics (2020) 40(2):354–75. doi: 10.1148/rg.2020190091 31951512

[12] Cherlin S Plant D Taylor JC Colombo M Cordell HJ. Prediction of Treatment Response in Rheumatoid Arthritis Patients Using Genome-Wide SNP Data. Genet Epidemiol (2018) 42(8):754–71. doi: 10.1002/gepi.22159 PMC6334178 30311271

[13] Ding D Pei W Chen W Zuo Y Ren S. Analysis of Clinical Characteristics, Diagnosis, Treatment and Prognosis of 46 Patients With Primary Gastrointestinal Non-Hodgkin Lymphoma. Mol Clin Oncol (2014) 2(2):259–64. doi: 10.3892/mco.2013.224 PMC3917777 24649343

[14] Makharia GK Srivastava S Das P Goswami P Singh U Tripathi M. Clinical, Endoscopic, and Histological Differentiations Between Crohn’s Disease and Intestinal Tuberculosis. Am J Gastroenterol (2010) 105(3):642−51. doi: 10.1038/ajg.2009.585 20087333

[15] Daum S Ullrich R Heise W Dederke B Foss HD Stein H. Intestinal Non-Hodgkin’s Lymphoma: A Multicenter Prospective Clinical Study From the German Study Group on Intestinal Non-Hodgkin’s Lymphoma. J Clin Oncol (2003) 21(14):2740–46. doi: 10.1200/JCO.2003.06.026 12860953

[16] Sun ZH Zhou HM Song GX Zhou ZX Bai L. Intestinal T-Cell Lymphomas: A Retrospective Analysis of 68 Cases in China. World J Gastroenterol (2014) 20(1):296–302. doi: 10.3748/wjg.v20.i1.296 24415885 PMC3886022

[17] Ding W Zhao S Wang J Yang Q Sun H Yan J. Gastrointestinal Lymphoma in Southwest China: Subtype Distribution of 1,010 Cases Using the WHO (2008) Classification in a Single Institution. Acta Haematol (2016) 135(1):21–8. doi: 10.1159/000437130 26303279

[18] Feuerstein JD Cheifetz AS. Crohn Disease: Epidemiology, Diagnosis, and Management. Mayo Clin Proc (2017) 92(7):1088–103. doi: 10.1016/j.mayocp.2017.04.010 28601423

[19] Liu YY Chen MK Cao Z Liu SZ Ding BJ. Differential Diagnosis of Intestinal Tuberculosis From Crohn’s Disease and Primary Intestinal Lymphoma in China. Saudi J Gastroenterol (2014) 20(4):241–7. doi: 10.4103/1319-3767.136979 PMC4131307 25038210

[20] Choi D Lim HK Lee SJ Lim JH Kim SH Lee WJ. Gastric Mucosa-Associated Lymphoid Tissue Lymphoma: Helical CT Findings and Pathologic Correlation. AJR Am J Roentgenol (2002) 178(5):1117–22. doi: 10.2214/ajr.178.5.1781117 11959712

[21] Lewis RB Mehrotra AK Rodriguez P Manning MA Levine MS. From the Radiologic Pathology Archives: Gastrointestinal Lymphoma: Radiologic and Pathologic Findings. Radiographics (2014) 34(7):1934–53. doi: 10.1148/rg.347140148 25384294

[22] Bodily KD Fletcher JG Solem CA Johnson CD Fidler JL Barlow JM. Crohn Disease: Mural Attenuation and Thickness at Contrast-Enhanced CT Enterography–Correlation With Endoscopic and Histologic Findings of Inflammation. Radiology (2006) 238(2):505–16. doi: 10.1148/radiol.2382041159 16436815

[23] Meyers MA McGuire PV. Spiral CT Demonstration of Hypervascularity in Crohn Disease: "Vascular Jejunization of the Ileum" or the "Comb Sign". Abdom Imaging (1995) 20(4):327–32. doi: 10.1007/bf00203365 7549737

[24] Chen CY Jaw TS Wu DC Kuo YT Lee CH Huang WT. Am J Roentgenol (2010) 195(5):1124–30. doi: 10.2214/ajr.09.3129 20966317

[25] Kim HJ Ha HK Kim HJ Byeon JS Kim MJ Lee SS. Gastrointestinal Dissemination of Mucosa-Associated Lymphoid Tissue Lymphoma: Computed Tomographic Findings. J Comput Assist Tomogr (2010) 34(2):187–92. doi: 10.1097/RCT.0b013e3181bbd21e 20351501

[26] Hanley JA McNeil BJ. A Method of Comparing the Areas Under Receiver Operating Characteristic Curves Derived From the Same Cases. Radiology (1983) 148(3):839–43. doi: 10.1148/radiology.148.3.6878708 6878708

[27] Wasserstein RL Schirm AL Lazar NA. Moving to a World Beyond P < 0.05. Am Stati (2019) 73(sup1):1–19. doi: 10.1080/00031305.2019.1583913

